# Bacterial profile and antimicrobial susceptibility patterns of common neonatal sepsis pathogens in Gulf Cooperation Council countries: A systematic review and meta-analysis

**DOI:** 10.5339/qmj.2024.62

**Published:** 2024-11-11

**Authors:** Muhammad Candragupta Jihwaprani, Idris Sula, Daniel Coha, Ahmed Alhebshi, Mohamad Alsamal, Ahmad M. Hassaneen, Mateq Ali Alreshidi, Nazmus Saquib

**Affiliations:** 1 Department of Pediatrics, Sidra Medicine, Doha, Qatar; 2 College of Medicine, Sulaiman Al Rajhi University, Al Bukaryiah, Saudi Arabia *Email: a.saquib@sr.edu.sa; 3 Department of Internal Medicine, Sulaiman Al Habib Medical, Group, Buraydah, Saudi Arabia; 4 Department of Medical Laboratory Sciences, College of Applied Sciences, Sulaiman Al Rajhi University, Al Bukaryiah, Saudi Arabia; 5 Department of Clinical Pathology, Faculty of Medicine, Zagazig University, Zagazig, Egypt

**Keywords:** Neonatal sepsis, newborn sepsis, bacterial resistance, antibiotic resistance, Gulf Cooperation Council, Bahrain, Kuwait, Oman, Qatar, Saudi Arabia, United Arab Emirates

## Abstract

**Introduction:**

Neonatal sepsis (NS) is a major healthcare burden in Gulf Cooperation Council (GCC) countries, with a prevalence higher than the global average. Microbial drug resistance has major implications for mortality and morbidity from NS.

**Objective:**

To synthesize data regarding the patterns of causative bacteria of NS in the GCC and their antimicrobial susceptibility profiles.

**Methods:**

Following the exploration of four electronic databases, i.e., EBSCOhost, ProQuest, PubMed/MEDLINE, and ScienceDirect, eligible studies were identified (i.e., published between 2013 and 2023 and reported bacterial profile and/or antimicrobial susceptibility patterns). The outcomes included the pooled prevalence of bacteria and their susceptibility patterns. Proportion meta-analysis was performed for each outcome of interest.

**Results:**

Fifteen studies were eligible (total positive cases = 2,473). Coagulase-negative *Staphylococci* (CoNS) (28.1%) were the most common gram-positive causative pathogen, followed by group B *Streptococcus* (GBS) (16.2%) and *Staphylococcus aureus* (9.9%); for gram-negative, *Escherichia coli* (12.7%) and *Klebsiella* species (11.4%) were most common. The susceptibility rates of these bacteria to first-line antibiotics were high; gram-positive bacteria had the highest susceptibility to ampicillin (72.8–98%), and gram-negative bacteria was most susceptible to amikacin (94.6–98%). Additionally, both gram-positive (67–77%) and negative (87–93%) bacteria exhibited high susceptibility to gentamicin.

**Conclusion:**

The most common pathogens among NS patients were gram-positive. The pathogens, irrespective of stain test, were susceptible to the current antibiotic therapy. We recommend the judicious use of empirical antibiotic therapy to prevent the growing risk of antimicrobial resistance.

## 1. Introduction

Neonatal sepsis (NS) is a bloodstream infection that can affect newborns within the first 28 days of life. It is classified into early-onset NS (EONS) if it occurs within ≤72 hours of life and late-onset NS (LONS) if it occurs after 72 hours. The incidence of NS has increased globally by 12.79% in the last three decades, from 5.6 million cases in 1990 to 6.3 million cases in 2019.^1^ In Gulf Cooperation Council (GCC) countries, a 2-year retrospective study found the incidence of regional EONS and LONS was 1.5/1,000 births and 11.63/1,000 births, respectively.^2,3^ These are higher compared to the global incidence of 0.97/1,000 births. According to some estimates, around 48,000 culture-proven cases of LONS occur annually in Kuwait, Saudi Arabia, and the United Arab Emirates (UAE), resulting in more than 1,000 deaths.^2,3^ Moreover, NS remains the third most common cause of death among neonates^4^ despite a steady reduction of mortality from it globally.^1^ Importantly, the reduction in NS mortality has been the slowest among other causes of death in neonates.^5^ The mortality and morbidity burden from NS is highest among developing countries, ranging from 23% to 50%.^6^

Drug resistance has major implications for mortality and morbidity from NS. A study in Jordan found that NS due to the most resistant organisms (*Acinetobacter baumannii* and *Klebsiella pneumoniae* carbapenemase-producing bacteria) was associated with higher mortality.^7^ Similar findings were also reported in Taiwan, where they found that an infection by multidrug-resistant bacteria within 7 days of admission to the neonatal intensive care unit (NICU) was significantly associated with higher mortality.^8^ Other risk factors associated with mortality include EONS, prematurity, neutropenia, thrombocytopenia, infection by gram-negative bacteria, low birth weight (<1,000 gram), and delay in administering appropriate antibiotic therapy.^6-9^

A recent systematic review of EONS in the Middle East and North Africa (MENA) found different causative pathogens, particularly *Klebsiella* species, group B *Streptococcus* (GBS), *Escherichia coli*, etc.^10^ Antibiotic susceptibility was also different across MENA countries, with better susceptibility profiles among high-income countries.^10^ However, that systematic review focused only on EONS even though LONS accounts for more than 70% of overall cases.^1^ Considering the diversity of the MENA region and the differences in socioeconomics and health, more focus is needed on individual areas to specify regional guidelines and recommendations. In the Middle East region, the countries of the GCC (Bahrain, Kuwait, Oman, Qatar, Saudi Arabia, and the UAE) have similar socioeconomics, geography, demographics, and health policies and standards, which is distinguishable from the rest of the MENA regions. We identified a gap in the literature concerning a meta-analytical review of NS pathogens specific to GCC countries, which warranted a comprehensive overview of this issue. Therefore, we conducted a systematic review and meta-analysis focusing on the patterns of NS pathogenic bacteria and their antimicrobial susceptibility profiles in GCC countries.

## 2. Methodology

### 2.1. Protocol and registration

This meta-analysis was conducted in compliance with the Preferred Reporting Items for Systematic Reviews and Meta-analyses (PRISMA) protocol and was prospectively registered at the International Prospective Register of Systematic Reviews (PROSPERO) with the identification number CRD42024498068.

### 2.2. Literature search strategy and eligibility criteria

We selected four electronic databases, i.e., EBSCOhost, ProQuest, PubMed/MEDLINE, and ScienceDirect, to retrieve all eligible studies. The following search terms were used: ((NS) OR (newborn sepsis) OR (neonatal bacteremia) OR (newborn bacteremia)) AND ((GCC) OR (Bahrain) OR (Kuwait) OR (Oman) OR (Qatar) OR (Saudi Arabia) OR (UAE)); they were individualized according to the search languages. All available studies from January 2013 through December 2023 were identified in the databases. Two reviewers (IS and AA) independently assessed the retrieved records for duplication and compliance with eligibility criteria. Discrepancies between reviewers at each stage were resolved by a third reviewer (MCJ).

### 2.3. Eligibility criteria

Observational studies carried out in any GCC country (Bahrain, Kuwait, Oman, Qatar, Saudi Arabia, and the UAE) that reported on the bacterial profile and/or antimicrobial susceptibility patterns of either EONS or LONS and published in English were included. Review articles, commentaries, letters to the editor, and those without clear results were excluded.

### 2.4. Data extraction

Following the full-text review of screened studies, data were extracted onto a predefined Excel spreadsheet. A data extraction tool was prepared that included the following variables: name of principal author, publication year, country, healthcare setup, sample size, study design, study period, sex of study participants, onset of the NS (EONS and LONS), types and number of bacteria isolates, and antibiotic resistance pattern. Data extraction was conducted by IS, AH, and MJ.

### 2.5. Quality assessment

Individual included studies were assessed for quality using the Newcastle-Ottawa Scale (NOS) for either cohort studies or cross-sectional studies.^11,12^ The assessment was done by two independent reviewers (MJ and IS). The tool evaluates three domains in the observational studies: (1) selection (sample representativeness, non-respondents, and exposure measurement), (2) comparability, and (3) outcome (outcome assessment method and statistical analysis). A study with a cumulative score of ≥7 was regarded as a good quality study.^11^

### 2.6. Outcome definitions

This meta-analysis assessed two outcomes for a 10-year span in six GCC countries: (1) the overall prevalence of bacterial etiology of NS, and (2) the overall antibiotic susceptibility pattern of each causative pathogen of NS.

### 2.7. Statistical analysis

Data synthesis and statistical analysis were performed by using OpenMeta [Analyst] software, Brown University. The prevalence of antibiotic resistance was computed using absolute numbers reported by each study. Continuous data on pooled prevalence and antibiotic sensitivity patterns were reported as proportions (%). A random-effects analysis model was selected given the high variability among included studies. Heterogeneity was evaluated by Cochrane *I*^2^ statistics. A *p*-value of <0.05 was considered significant at a two-tailed 95% confidence interval (CI).

## 3. Results

### 3.1. Study selection

**Figure 1.**
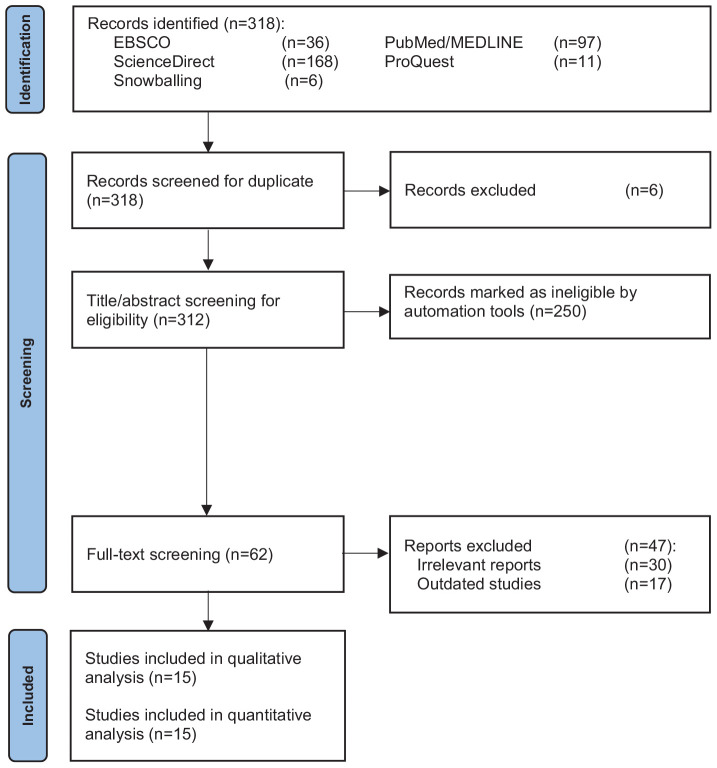
PRISMA flow diagram of included studies.

The database searches retrieved 316 records; six duplicate records were removed. An additional 231 records were excluded after title/abstract screening, followed by 62 records after full-text review, leaving 15 studies eligible for this systematic review and meta-analysis (Figure 1).

### 3.2. Characteristics of studies included in the meta-analysis

**Figure 2.**
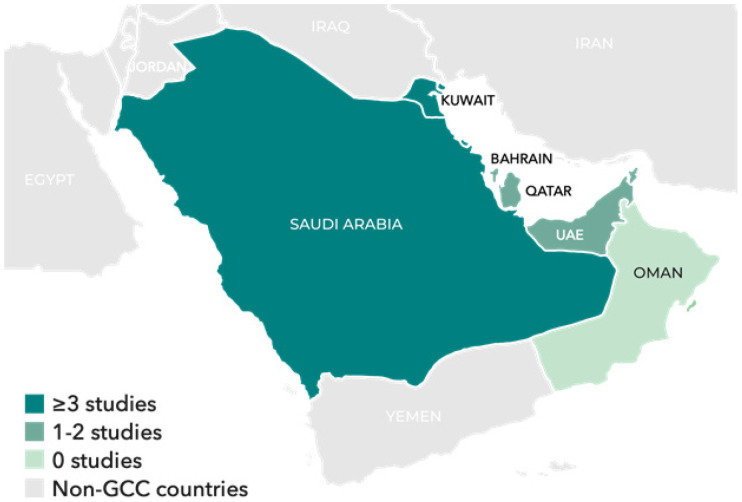
Number of studies included from each Gulf Cooperation Council country. GCC: Gulf Cooperation Council; UAE: the United Arab Emirates.

All included studies were published between 2013 and 2023. Six studies were from Saudi Arabia, two from Qatar, and one from Bahrain. Two were multi-country studies (Figure 2). Most studies used a retrospective study design (*n* = 9 [retrospective cohort = 7 and prospective cohort = 2]), and all studies examined results of blood cultures from suspected cases of neonatal septicemia.

### 3.3. Characteristics of the study population

A total of 2,473 cases were reported (Table 1). Most studies reported the sex distribution of their included study participants (*n* = 12); 1,167 study participants were boys and 995 were girls. The majority of included cases were LONS (*n* = 1,475).

### 3.4. Meta-analysis for the prevalence of bacteria isolates from neonatal sepsis in GCC countries

A total of 15 bacterial species were reported as causative pathogens of NS in GCC countries (Table 2 and Supplementary Table 1). The most prevalent gram-positive bacteria were coagulase-negative *Staphylococci* (CoNS) (pooled prevalence = 28.1% [95% CI = 17.9–38.4]), followed by *Staphylococcus* spp. (19.1% [95% CI = 3.3–41.5]), GBS (16.2% [95% CI = 5.4–22.8]), and *Staphylococcus* aureus (9.9% [95% CI = 5.3–14.6]). The most prevalent gram-negative bacteria were *E. coli* and *Klebsiella* spp., with a pooled prevalence of 12.7% (95% CI = 8.9–16.4) and 11.4% (95% CI = 6.9–15.9), respectively.

### 3.5. The pooled effect size of antibiotic susceptibility patterns

Among CoNS isolates, the highest pooled susceptibility rate was to gentamicin (77.3% [95% CI = 66.3–88.3]), followed by ampicillin (72.8% [95% CI = 35.4–100]) (Table 3 and Supplementary Tables 2, 3). Among gram-negative bacteria, *K. pneumoniae* and *E. coli* were 94.6% (95% CI = 91.2–98) and 97.6% (95% CI = 94.5–100) susceptible to amikacin, respectively (Table 3 and Supplementary Figures 2, 3). These bacteria also showed a higher susceptibility rate to gentamicin and meropenem. *K. pneumoniae* showed the lowest susceptibility rate to ampicillin at 22.8% (95% CI = 13.8–59.3).

### 3.6. Quality of studies

The NOS quality assessment was applied to cross-sectional (*n* = 6) and cohort (*n* = 9) studies. Among the six cross-sectional studies (Table 4A), the majority were of fair quality (*n* = 3),^13-15^ two studies were of poor quality,^16,17^ and only one was of good quality.^18^ Quality assessment of the nine cohort studies (Table 4B) found that four were good,^2,19–21^ four were fair,^22-25^ and one study was of poor quality.^26^

## 4. Discussion

This meta-analysis aimed to assess the prevalence of pathogenic bacteria of NS and their antimicrobial susceptibility patterns in the GCC region. Fifteen studies from six GCC countries were pooled, and the data of fifteen isolated bacterial species were analyzed. We found that the most common isolated NS pathogens were gram-positive bacteria: CoNS (28.1%), *Staphylococcus* spp. (19.1%), and GBS (16.2%). Among gram-negative bacteria, *E. coli* accounted for most of the isolated pathogens (12.7%), followed by *Klebsiella spp.* (11.4%). Furthermore, the antimicrobial susceptibility tests revealed that common gram-positive species were mostly sensitive to gentamicin and ampicillin (77.3% and 72.8%, respectively, among CoNS), whereas gram-negative bacteria were highly susceptible to amikacin, gentamicin, and meropenem (Supplementary Material).

Our findings of the most prevalent gram-positive and gram-negative pathogens in GCC countries are consistent with a recent review of EONS in ten MENA countries.^10^ However, the distribution of common bacterial isolates in MENA countries was higher than in our study, including GBS (26% vs. 16.2%) and *E. coli* (24% vs. 12.7%), presumably because they assessed only EONS and had a smaller sample size. Similarly, in 2019, the Ministry of Health of Saudi Arabia published a protocol for screening and managing suspected NS, including a list of the most common causative pathogens (CoNS, *E. coli*, *Klebsiella* spp., etc.),^27^ which is consistent with our findings regarding the incidence of NS.

The susceptibility patterns found in our study revealed high sensitivity to the first-line regimens of NS. Our pooled susceptibility rates revealed very high susceptibility rates of gram-negative bacteria to amikacin and gentamicin, while gram-positive bacteria showed very high susceptibility rates to ampicillin. The American Academy of Pediatrics (AAP) and the British National Institute for Health and Care Excellence (NICE) guidelines recommend the empiric combination of beta-lactams (e.g., ampicillin) and aminoglycosides (e.g., gentamicin) for neonates with NS.^28,29^ In select cases, empiric use of vancomycin is recommended instead of ampicillin for gram-positive coverage (e.g., ampicillin-resistant CoNS, indwelling central catheter, and known colonization by methicillin-resistant *S. aureus* [MRSA]). Correspondingly, other gram-negative targeting agents may be alternatively used, such as third-/fourth-generation cephalosporins or carbapenems.^29^ These recommendations have also been included in the local/regional guidelines as empirical therapy.^27^

With regard to the MENA regions, a large systematic review done in July 2020 assessing the prevalence of EONS in ten MENA countries revealed similar susceptibility to first-line therapies, including ampicillin, gentamicin, and amikacin, especially in high-income MENA countries (93%).^10^ However, these rates were significantly lower in middle-income countries (40%), suggesting that low socioeconomic status might lead to lower susceptibility rates.^10^ By understanding the current regional antimicrobial susceptibility profiles of common NS causative pathogens as found in our meta-analysis, GCC physicians can create more precise and localized intervention strategies. Although the current empirical therapy remains effective, further specific therapies should be guided by the culture and sensitivity results. We also strongly encourage the judicious administration of antibiotics for neonatal infection to maintain and improve the susceptibility profiles of pathogenic bacteria.

### 4.1. Strengths and limitations

This is the first meta-analytical study to assess the prevalence and susceptibility profiles of pathogenic bacteria of NS in GCC countries. This study provides a holistic understanding of the current bacterial profile of NS in the region. The study comprehensively compiled available literature from a 10-year period to provide the best up-to-date evidence on the issue. Each pathogen was analyzed separately for susceptibility rates to different groups of antibiotics. The results were generally consistent with findings from other geographic areas, including MENA and Western countries. The implication of this study is considerable and can guide physicians in GCC countries on the appropriate empiric regimens for NS therapy.

There were a few limitations in our study. First, the study did not differentiate between the causative pathogens for EONS vs. LONS, as most of the studies did not classify their reports of the causative pathogens under categories such as time of onset, gender, etc. Second, there was an uneven and disproportionate distribution of samples across countries, with the majority of studies being retrieved from Saudi Arabia and Kuwait (≥3 studies), while other countries had ≤2 studies. We only included relevant studies conducted within the past 10 years to get an updated overview of the condition. Despite its publication date, one study from Oman^30^ was excluded because it began prior to 2013, which left Oman underrepresented. Additionally, it should be mentioned that most of the included studies did not report multidrug resistance patterns. Consequently, we were unable to provide an analysis of the multidrug resistance patterns exhibited by bacterial isolates of NS. Future studies should focus on the prevalence of multidrug-resistant strains of NS pathogens and their pattern of resistance in the region.

## 5. Conclusion

Both gram-positive and gram-negative bacteria were associated with NS in the GCC. The most common causative pathogens were CoNS, *Staphylococcus* spp., *E. coli*, and *Klebsiella* spp. Our pooled analysis showed that these pathogens are very susceptible to empirical therapy drugs, particularly ampicillin, gentamicin, and amikacin.

### 5.1. Implications for policy and practice

Even though the susceptibility rates of the most common pathogens to empirical therapies were high, increased focus and investment are needed to improve the management and clinical outcome of NS. This may include continuous updates to treatment protocols and increased research on antimicrobial resistance patterns in the region to inform future policy decisions related to public health and infection control.

## Abbreviations

**Table:** 

CoNS	Coagulase-negative Staphylococci
EONS	Early-onset neonatal sepsis
GBS	Group B Streptococcus
GCC	Gulf Cooperation Council
LONS	Late-onset neonatal sepsis
MENA	Middle East and North Africa
NICU	Neonatal Intensive Care Unit
NOS	Newcastle Ottawa Scale
NS	Neonatal sepsis
UAE	the United Arab Emirates

## Authors’ Contributions

Conceptualization: MCJ and IS; methodology: MCJ and IS; software: MCJ and IS; validation, NS, AMH, MAA; database searching: MCJ and IS; reports screening: MCJ, IS, and AA; quality assessment: MCJ and IS; data extraction and analysis: MCJ, IS, and AA; writing—original draft preparation: IS, MCJ, AA, DC, and MSA; writing—review and editing: IS and MCJ; visualization: IS and MCJ; supervision: NS, AMH, and MAA. All authors have read and agreed to the final version of the manuscript.

## Acknowledgment

We thank Erin Strotheide for her editorial contributions.

## Conflicts of Interest Statement

The authors declare no conflict of interest.

## Ethical Declaration

No ethical approval was necessary as this was a secondary synthesis of published articles.

## Data Availability Statement

The data presented in this study are available upon reasonable request from the corresponding author.

## Supplementary Materials

### Appendix 1

**Supplementary Table 1.** Pathogen isolates of individual studies.

**Author, year**	**Positive cases**	***Acinetobacter* spp.**	**CoNS**	***E. coli***	***Enterobacter* spp.**	**GBS**	***H. influenzae***	***Klebsiella* spp.**	**MRSA**	***P. aeruginosa***	***Pseudomonas* spp.**	***S. aureus***	**Serratia spp.**	***Staphylococcus* spp.**	***Streptococcus* spp.**	**γ-hemolytic *streptococci***	**Other**
Al-Zahrani, 2015	32	1	3	5	2	3		7				2	1			1	7
Alrafiaah, 2016	43	1	15	5	1	3		3	1	4		4			2		
Almannaei, 2017	67		32			7						3					25
Fattah, 2017	320			72		43	23										205
Hammoud, 2017 (EONS)	102		6	13	1	61		4				2			3	2	10
Hammoud, 2017 (LONS)	785	36	272	38	31	3		179	11		35	23	15		2	26	114
Al-Matary, 2019	298			29	18	15		41		22				126	4		43
Al Mouqdad, 2019	36	3	21	2	3												7
Almudeer, 2020	126		14	37		21	2	6			5	7					36
Alarjani, 2021	123			19				20			5	33		11	35		
Al Matary, 2022	237		75	18				23									121
Alharbi, 2022	40	2	23	2	2	2		4	1	1	1				2		0
Vellamgot, 2022	65		3	11		29		3	2					4	4	2	7
Gad, 2023	175	9		17	9		3	29			26	43	20			3	19
Mubaraki, 2023	24	1	6	5	1	4		2	1			4					
Total	2473	53	470	273	68	191	27	321	16	27	72	121	36	141	52	34	594

3GC: third-generation cephalosporin; CoNS: coagulase-negative *Staphylococci*; EONS: early-onset neonatal sepsis; GBS: group B *Streptococcus*; LONS: late-onset neonatal sepsis; MRSA: methicillin-resistant *Staphylococcus aureus*; spp: species; TMP/SMX: trimethoprim/sulfamethoxazole; TZP: piperacillin/tazobactam.

**Supplementary Table 2.** Antimicrobial susceptibility rates of individual causative pathogens of neonatal sepsis across included studies.

**Author, year**	**Pathogens**	**Tested pathogens**	**3GC**	**Amikacin**	**Ampicillin**	**Cefepime**	**Cefotaxime**	**Ceftazidime**	**Ceftriaxone**	**Cloxacillin**	**Gentamicin**	**Imipenem**	**Meropenem**	**TMP/SMX**	**TZP**	**Vancomycin**
Hammoud, 2017	*Acinetobacter* spp.	35	12	33							33					
Maannaei, 2017	CoNS	32		21	17		17				23		15			
Alharbi, 2022		23		9	21		13		2	11	19		4		6	
Hammoud, 2017 (EONS)	*E. coli*	12		12	2						12					
Hammoud, 2017 (LONS)		35	32	33/34							33/34					
Al-Matary, 2019		29		29	4	18	18	17			18	29	29	14	25	
Aljarani, 2021		19		19	11				16		18			15		
Alharbi, 2022		2		2	2		2		1	2	2		1			
Hammoud, 2017	*Enterobacter* spp.	31	17	31							31					
Al-Matary, 2019		18		18	1	15	11	12			15	18	18	16	14	
Al-Matary, 2019	*Enteroccoccus fecalis*	6			5											6
Hammoud, 2017	GBS	48			48											
Maannaei, 2017		7			7		3				5					
Al-Matary, 2019		15														15
Alharbi, 2022		2		0	2		2		0	0	1				0	
Hammoud, 2017	*Klebsiella* spp.	161	71/161	92/100							92/100					
Al-Matary, 2019		40		39	0	33	33	34			33	40	40	33	38	
Aljarani, 2021		20		18	2				5		17			12		
Alharbi, 2022		4		4	2		2		1	4	4		3			
Alharbi, 2022	MRSA	1		1	0		1		0	1	0		1		0	
Hammoud, 2017	*Pseudomonas* spp.	34	26/34	31/33							31/33					
Al-Matary, 2019		22		22		22		17			22	20	20		22	
Aljarani, 2021		5			2				1		2			2		
Hammoud, 2017	*Serratia* spp.	14	10/14	10/11							10/11					
Al-Matary, 2019	*Staphylococcus* spp.	119												66		119
Aljarani, 2021	*Staphylococcus aureus*	33			19				22		20					16
Al-Matary, 2019	*Streptococcus* spp.	4			3									1		4
Aljarani, 2021		35			16				17		23					

3GC: third-generation cephalosporin; CoNS: coagulase-negative *Staphylococcus*; EONS: early-onset neonatal sepsis; GBS: group B *Streptococcus*; LONS: late-onset neonatal sepsis; MRSA: methicillin-resistant *Staphylococcus aureus*; spp: species; TMP/SMX: trimethoprim/sulfamethoxazole; TZP: piperacillin/tazobactam.

### Appendix 2

**Supplementary Table 3.** Pooled bacterial sensitivity prevalence of causative pathogens of neonatal sepsis in Gulf Cooperation Council countries.

					**Pooled estimation**		**Heterogeneity test**	
**Bacteria**	**Antibiotics**	**Number of studies**	**Number of isolates tested**	**Number of susceptible isolates**	**Pooled prevalence (%)**	**95% CI**	***I* ^2^**	***p*-value**
Acinetobacter spp.	3GC	1	35	12				
	Amikacin	1	35	33				
	Gentamicin	1	35	33				
CoNS	Ampicillin	3	55	38	72.8	35.4-100	92.23	0.000
	Ceftriaxone	3	78	32	38.8	4.9-72.6	92.45	0.000
	Amikacin	2	55	30	53	27.1-78.9	75.2	0.045
	Gentamicin	2	55	42	77.3	66.3-88.3	0.00	0.338
	Meropenem	2	55	19	31.9	3-60.8	83.86	0.013
	Cloxacillin	1	23	11	47.8			
	TZP	1	23	6	26.1			
*E. coli*	3GC	6	116	86	75	60.3-89.7	69.11	0.006
	Amikacin	5	96	95	97.6	94.5-100	0.00	0.958
	Gentamicin	5	96	86	89.9	79.8-100	71.61	0.007
	Ampicillin	4	62	19	38.6	11-66.2	84.55	0.000
	Meropenem	2	31	30	87	47-100	46.26	0.173
	TMP/SMX	2	48	29	63.6	33.5-93.6	0.00	0.02
	Cefepime	1	29	18	62.1	NA	NA	NA
	Cloxacillin	1	2	2	100	NA	NA	NA
	Imipenem	1	29	29	100	NA	NA	NA
	TZP	1	29	25	86.2	NA	NA	NA
*Enterococcus* spp.	Ampicillin	1	6	5				
	Vancomycin	1	6	6				
*Entorobacter* spp.	3GC	3	67	40	59.9	48.2-71.6	0.00	0.704
	Gentamicin	2	49	46	93.3	79.2-100	64.7	0.095
	Amikacin	1	31	31	100			
	Ampicillin	1	18	1	5.6			
	Cefepime	1	18	15	83.3			
	Imipenem	1	29	18	62.1			
	Meropenem	1	18	18	100			
	TMP/SMX	1	18	16	88.9			
	TZP	1	18	14	77.8			
GBS	3GC	3	15	5	29.1	3.1-55.1	36.62	0.206
	Ampicillin	3	57	57	98.8	96-100	0.00	0.644
	Gentamicin	2	9	6	67.4	37.2-97.5	0.00	0.585
	Meropenem	2	44	43	96.7	83.5-100	16.59	0.274
	Amikacin	1	2	0	0			
	Imipenem	1	40	40	100			
	TZP	1	2	0	0			
*Klebsiella* spp.	3GC	6	269	146	54.6	31.9-77.2	92.42	0.000
	Amikacin	4	164	153	94.6	91.2-98	0.00	0.409
	Gentamicin	4	164	144	86.9	78.7-95.1	40.24	0.17
	Ampicillin	2	24	4	22.8	13.8-59.3	58.12	0.122
	Meropenem	2	44	43	96.7	83.5-100	16.59	0.274
	TMP/SMX	2	60	45	73.1	51.4-94.9	69.17	0.072
	Cefepime	1	40	33	82.5			
	Cloxacillin	1	4	4	100			
	Imipenem	1	40	40	100			
	TZP	1	40	38	95			
MRSA	3GC (Cefotaxime)	1	1	1				
	3GC (Ceftriaxone)	1	1	0				
	Amikacin	1	1	1				
	Ampicillin	1	1	0				
	Cloxacillin	1	1	1				
	Gentamicin	1	1	0				
	Meropenem	1	1	1				
	TZP	1	1	0				
*Pseudomonas* spp.	3GC	3	61	44	63.2	38-88.4	78.19	0.010
	Amikacin	2	56	53	95.6	89.4-100	25.58	0.246
	Ampicillin	1	5	2				
	Cefepime	1	22	17				
	Gentamicin	3	60	55	92.2	80.4-100	71.95	0.028
	Imipenem	1	22	20				
	Meropenem	1	22	20				
	TMP/SMX	1	5	2				
	TZP	1	22	22				
*S. aureus*	3GC	1	33	22				
	Ampicillin	1	33	19				
	Gentamicin	1	33	20				
	Vancomycin	1	33	16				
*Serratia* spp.	3GC	1	14	10				
	Amikacin	1	11	10				
	Gentamicin	1	11	10				
*Staphylococcus* spp.	TMP/SMX	1	119	66				
	Vancomycin	1	119	119				

3GC: third-generation cephalosporin; CoNS: coagulase-negative *Staphylococci*; GBS: group B *Streptococcus*; MRSA: methicillin-resistant *Staphylococcus* aureus; spp, species; TMP/SMX: trimethoprim/sulfamethoxazole; TZP: piperacillin/tazobactam.

### Appendix 3

**Supplementary Figure 1.**
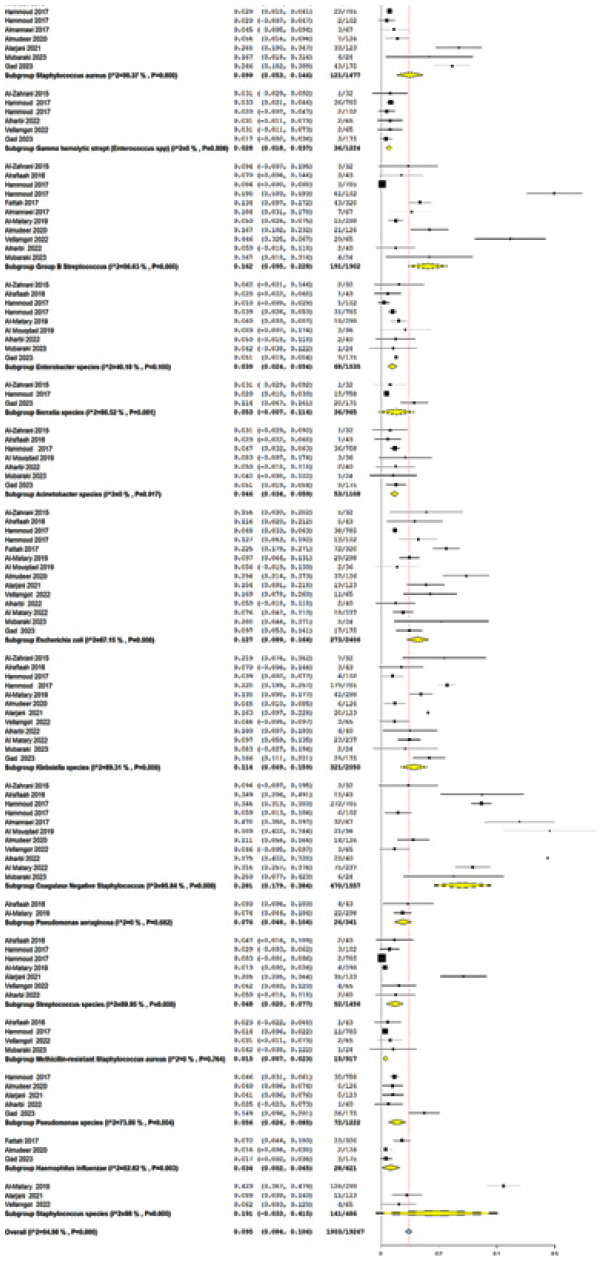
Forest plot of the pooled prevalence of isolated pathogens.

**Supplementary Figure 2.**
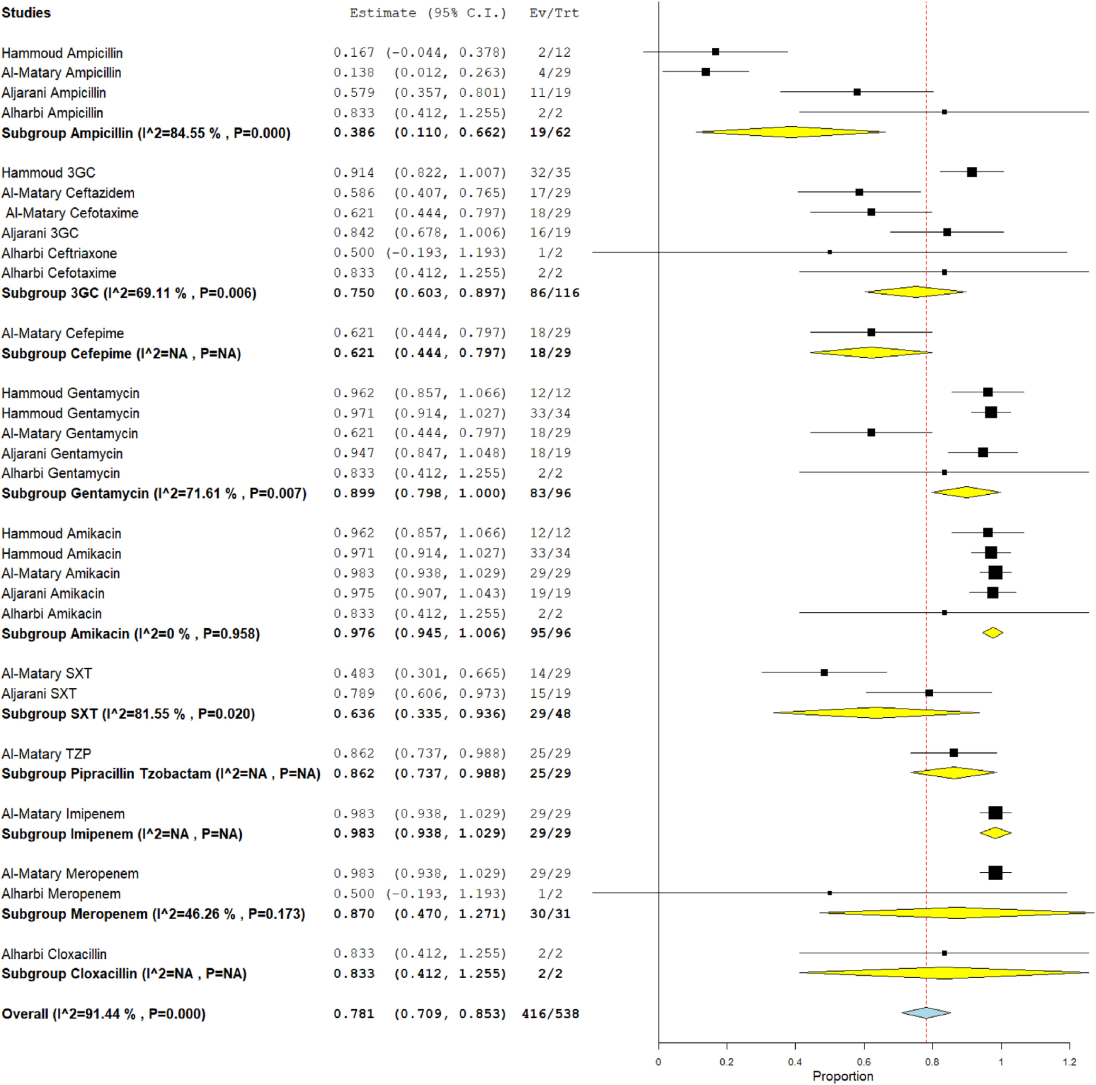
Forest plot of the pooled susceptibility pattern of *Escherichia coli*.

**Supplementary Figure 3.**
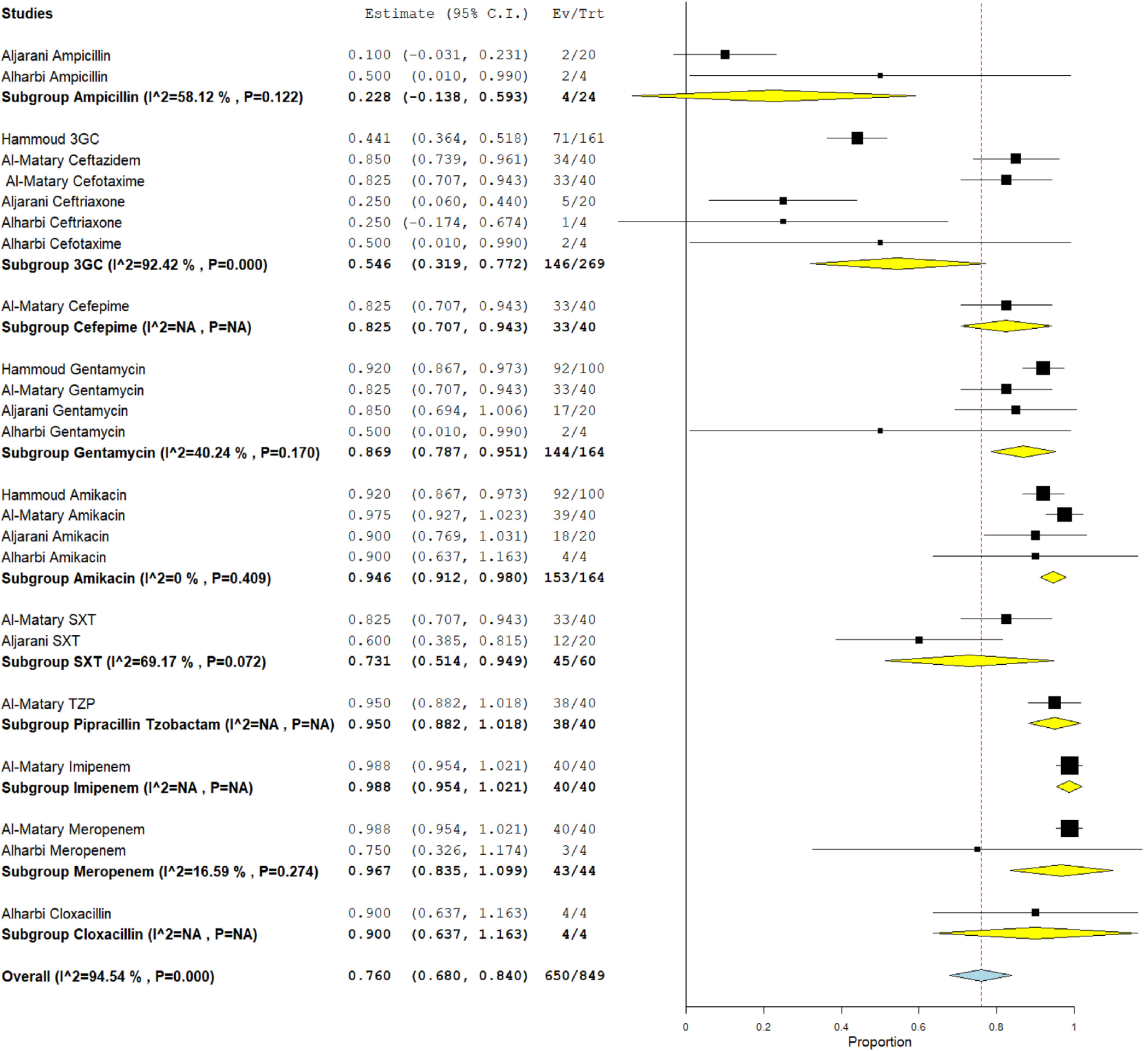
Pooled susceptibility pattern of *Klebsiella pneumoniae*.

**Supplementary Figure 4.**
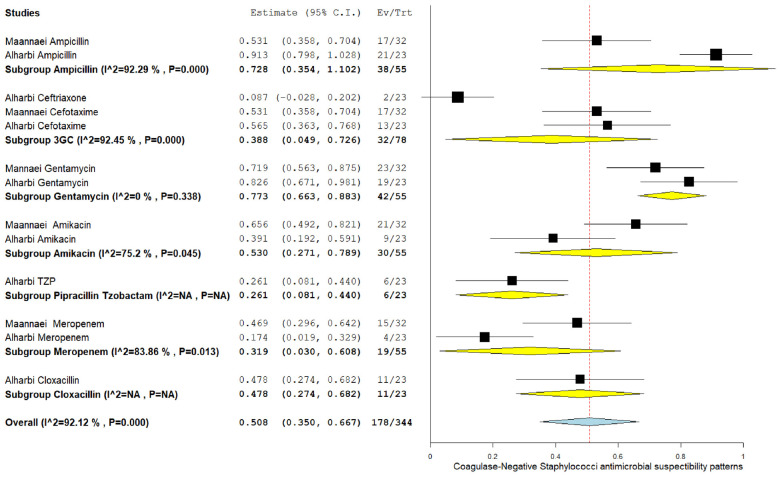
Forest plot of pooled susceptibility pattern of coagulase-negative *Staphylococcus* spp.

**Supplementary Figure 5.**
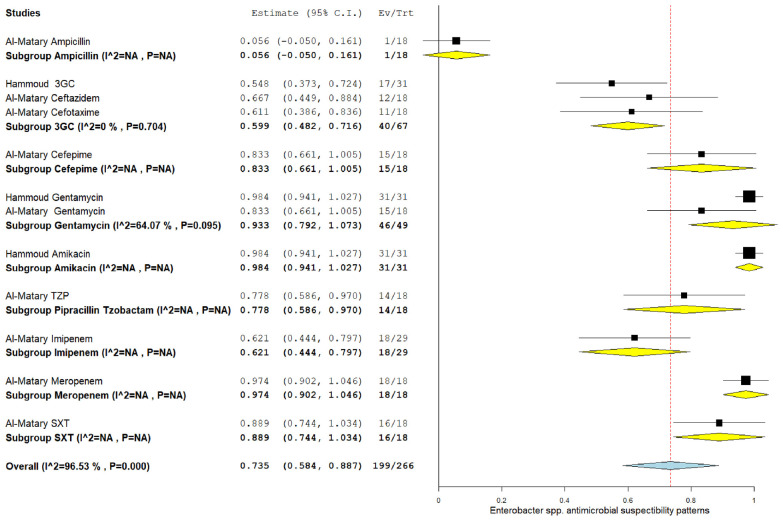
Pooled susceptibility pattern of *Enterobacter* spp.

**Supplementary Figure 6.**
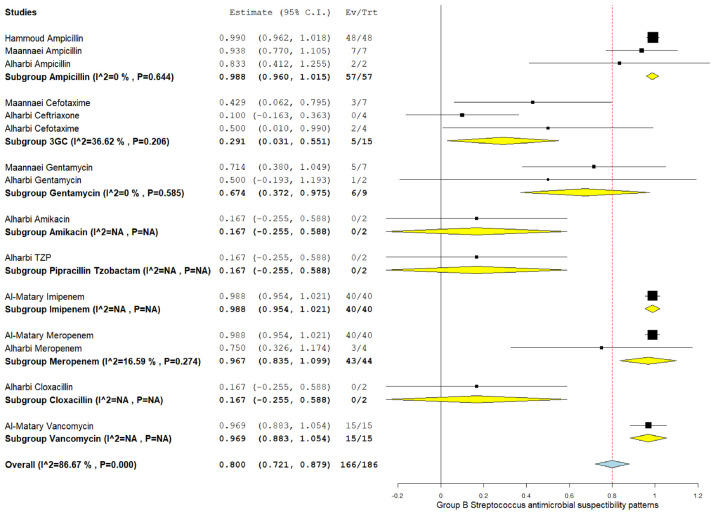
Forest plot of pooled susceptibility pattern of group B *Streptococcus*.

**Supplementary Figure 7.**
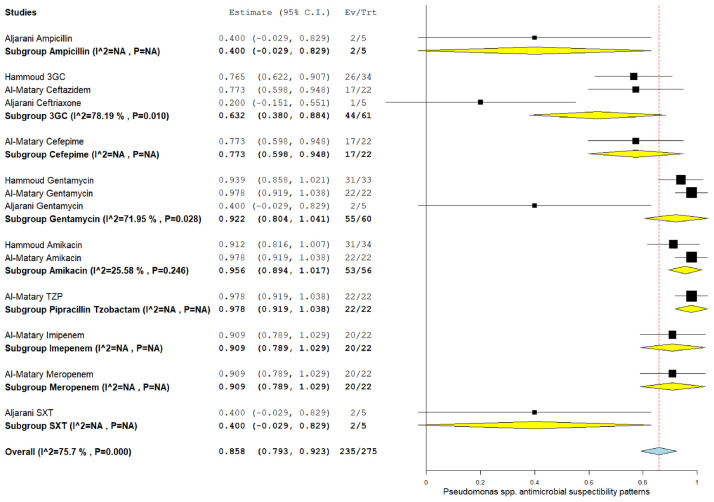
Forest plot of pooled susceptibility pattern of *Pseudomonas* spp.

## Figures and Tables

**Table 1.** Characteristics of included studies.

**Author, year**	**Study design**	**Study period**	**Country (city)**	**Healthcare setup**	**Positive cases**	**M**	**F**	**EONS (*n*)**	**LONS (*n*)**
Al-Zahrani, 2015	CS	Jan 2013–Jan 2014	Saudi Arabia (Taif)	King Abdulaziz Specialist Hospital	32	NR	NR	32	NR
Alrafiaah, 2016	CS	Jan–Dec 2014	Saudi Arabia (Riyadh)	King Abdullah International Medical Research Center	43	25	18	17	26
Almannaei, 2017	CS	Jul 2013–Sep 2015	Bahrain (Manama)	King Hamad University Hospital	67	34	33	18	49
Fattah, 2017	CS	Jan 2013–Aug 2015	Saudi Arabia (Riyadh)	King Abdulaziz Medical City	320	81	79	80	80
Hammoud, 2017 (EONS)	PC	Jun 2013–May 2015	Kuwait (Kuwait city), UAE (Dubai), Saudi Arabia (Jeddah)	Al Sabah Maternity Hospital (Kuwait), Dubai Hospital and Tawam Hospital (UAE); King Abdulaziz Hospital and the Maternity and Children’s Hospital (Saudi Arabia)	102	50	52	102	NR
Hammoud, 2017 (LONS)	PC	Jun 2013–May 2015	Kuwait (Kuwait city), UAE (Dubai), Saudi Arabia (Jeddah)	Al Sabah Maternity Hospital (Kuwait), Dubai Hospital and Tawam Hospital (UAE); King Abdulaziz Hospital and the Maternity and Children’s Hospital (Saudi Arabia)	785	413	372	NR	785
Al-Matary, 2019	RC	Jan 2011–Dec 2015	Saudi Arabia (Riyadh)	King Fahad Medical City	298	168	130	33	265
Al Mouqdad, 2019	RC	Jan 2014–Dec 2017.	Saudi Arabia (Riyadh)	King Saud University Medical City	36	NR	NR	0	36
Almudeer, 2020	RC	May 2012–Apr 2019	Saudi Arabia (Jazan)	King Fahad Central Hospital	126	65	61	NR	NR
Alarjani, 2021	CS	NR	Saudi Arabia (Riyadh)	King Saud University Medical City	123	52	71	49	74
Alharbi, 2022	RC	May 2011–Oct 2018	Saudi Arabia (Jeddah)	King Abdulaziz University Hospital	40	19	21	NR	NR
Al Matary, 2022	RC	Jan 2016–May 2020	Saudi Arabia (Riyadh)	King Fahad Medical City	237	125	112	NR	NR
Vellamgot, 2022	RC	Jan 2016–Dec 2019	Qatar (Doha)	Al-Wakra Hospital	65	NR	NR	NR	NR
Gad, 2023	RC	Jan 2015–Dec 2019	Qatar (Doha)	Hamad Medical Corporation	175	123	52	NR	175
Mubaraki, 2023	CS	Sep 2019–Aug 2020	Saudi Arabia (Riyadh)	King Saud University Medical City	24	12	12	13	11
Total					2473	1167	995	295	1475

CS: cross-sectional; EONS: early-onset neonatal sepsis; F: female; LONS: late-onset neonatal sepsis; M: male; NR: not reported; PC: prospective cohort; RC: retrospective cohort.

**Table 2.** Prevalence of causative pathogens of neonatal sepsis.

				Pooled estimation*		**Heterogeneity test**	
**Bacteria**	**Number of studies**	**Total organisms**	**Number of isolates**	**Pooled prevalence**	**95% CI**	***I* ^2^**	***p*-value**
*Acinetobacter spp.*	7	1108	53	4.6	3.4–5.9	0	0.917
CoNS	11	1557	470	28.1	17.9–38.4	95.84	0.0001
*E. coli*	14	2406	273	12.7	8.9–16.4	87.15	0.0001
*Enterobacter* spp.	9	1535	68	3.9	2.4–5.4	40.18	0.1
Gamma-hemolytic *streptococci*	5	1159	34	2.7	1.8–3.7	0	0.689
GBS	11	1902	191	16.2	5.4–22.8	96.63	0.0001
*H. influenzae*	3	621	28	3.4	0.2–6.5	82.82	0.003
*Klebsiella* spp.	12	2050	321	11.4	6.9–15.9	89.31	0.0001
MRSA	4	914	15	1.5	0.07–2.3	0	0.756
*P. aeruginosa*	2	341	26	7.6	4.8–10.4	0	0.682
*Pseudomonas* spp.	5	1222	72	5.6	2.6–8.5	73.86	0.004
*S. aureus*	9	1477	121	9.9	5.3–14.6	99.37	0.0001
*Serratia* spp.	3	965	36	5.3	0.7–11.4	86.52	0.001
*Staphylococcus* spp.	3	486	141	19.1	3.3–41.5	98	0.000
*Streptococcus* spp.	7	1456	52	4.8	2–7.7	89.95	0.0001

CoNS: coagulase-negative *Staphylococci*; GBS: group B *Streptococcus*; MRSA: methicillin-resistant *Staphylococcus aureus*; spp.: species.

* The full plot for the pooled prevalence is available in the Supplementary Figure 1.

**Table 3.** The pooled bacterial susceptibility pattern of neonatal sepsis causative pathogens in Gulf Cooperation Council countries reported by ≥2 studies*

					Pooled estimation**		**Heterogeneity test**	
**Bacteria**	**Antibiotics**	**Number of studies**	**Number of isolates tested**	**Number of susceptible isolates**	**Pooled prevalence (%)**	**95% CI**	***I* ^2^**	***p*-value**
CoNS	Ampi cillin	3	55	38	72.8	35.4–100	92.23	0.000
	Ceftriaxone	3	78	32	38.8	4.9–72.6	92.45	0.000
	Amikacin	2	55	30	53	27.1–78.9	75.2	0.045
	Gentamicin	2	55	42	77.3	66.3–88.3	0.00	0.338
	Meropenem	2	55	19	31.9	3–60.8	83.86	0.013
*E. coli*	3GC	6	116	86	75	60.3–89.7	69.11	0.006
	Amikacin	5	96	95	97.6	94.5–100	0.00	0.958
	Gentamicin	5	96	86	89.9	79.8–100	71.61	0.007
	Ampicillin	4	62	19	38.6	11–66.2	84.55	0.000
	Meropenem	2	31	30	87	47–100	46.26	0.173
	TMP/SMX	2	48	29	63.6	33.5–93.6	0.00	0.02
*Entorobacter* spp.	3GC	3	67	40	59.9	48.2–71.6	0.00	0.704
	Gentamicin	2	49	46	93.3	79.2–100	64.7	0.095
GBS	3GC	3	15	5	29.1	3.1–55.1	36.62	0.206
	Ampicillin	3	57	57	98.8	96–100	0.00	0.644
	Gentamicin	2	9	6	67.4	37.2–97.5	0.00	0.585
	Meropenem	2	44	43	96.7	83.5–100	16.59	0.274
*Klebsiella* spp.	3GC	6	269	146	54.6	31.9–77.2	92.42	0.000
	Amikacin	4	164	153	94.6	91.2–98	0.00	0.409
	Gentamicin	4	164	144	86.9	78.7–95.1	40.24	0.17
	Ampicillin	2	24	4	22.8	13.8–59.3	58.12	0.122
	Meropenem	2	44	43	96.7	83.5–100	16.59	0.274
	TMP/SMX	2	60	45	73.1	51.4–94.9	69.17	0.072
*Pseudomonas* spp.	3GC	3	61	44	63.2	38–88.4	78.19	0.010
	Gentamicin	3	60	55	92.2	80.4–100	71.95	0.028
	Amikacin	2	56	53	95.6	89.4–100	25.58	0.246

3GC: third-generation cephalosporins; CoNS: coagulase-negative Staphylococci; GBS: group B Streptococcus; MRSA: methicillin-resistant Staphylococcus aureus; spp.: species; TMP/SMX: trimethoprim/sulfamethoxazole; TZP: piperacillin/tazobactam.

* The full table is available in the Supplementary Table 3).

** The plots for the pooled susceptibility rates are available in the Supplementary Figures 2–7.

**Table 4.** Quality assessment of (A) included cross-sectional studies using the Newcastle-Ottawa tool adapted for cross-sectional studies, and (B) included cohort studies using the Newcastle-Ottawa tool for cohort studies. (A) Cross-sectional studies assessment.

	**Selection**			**Comparability**	**Outcome**			
**Author, year**	**Representativeness of the sample**	**Non-respondents**	**Ascertainment of the exposure (risk factor)**	**Comparability between groups**	**Assessment of outcome**	**Statistical test**	**Total**	**Quality of study**
Al Zahrani, 2015	*	0	*	*	*	*	5	Fair
Alrafiaah, 2016	*	0	*	**	*	0	5	Fair
Almannaei, 2017	0	0	*	0	*	0	2	Poor
Fattah, 2017	*	0	*	**	*	*	6	Fair
Alarjani, 2021	0	*	*	0	*	0	3	Poor
Mubaraki, 2023	*	*	*	**	*	*	7	Good

**Table:** (B) Cohort studies assessment.

	**Selection**				**Comparability**	**Outcome**				
**Author, year**	**Representativeness of the sample**	**Selection of the non-exposed cohort**	**Ascertainment of the exposure (risk factor)**	**Demonstration that outcome of interest was not present at start of study**	**Comparability between groups**	**Assessment of outcome**	**Was follow-up long enough for outcomes to occur**	**Adequacy of follow up of cohorts**	**Total**	**Quality of study**
Hammoud, 2017 (EONS)	*	*	*	*	*	*	*	*	8	Good
Hammoud, 2017 (LONS)	*	*	*	*	*	*	*	*	8	Good
Al Matary, 2019	0	0	*	0	*	*	0	*	4	Poor
Al Mouqdad, 2019	0	*	*	0	*	*	0	*	5	Fair
Almudeer, 2020	*	*	*	0	*	*	*	*	7	Good
Alharbi, 2022	0	*	*	*	0	*	*	0	5	Fair
Al Matary, 2022	0	*	*	0	*	*	0	*	5	Fair
Vellamgot, 2022	*	*	*	0	**	*	*	*	8	Good
Gad, 2023	0	*	*	0	*	*	0	*	5	Fair
